# Clinical Characteristics and Multisystem Imaging Findings of COVID-19: An Overview for Orthopedic Surgeons

**DOI:** 10.1007/s11420-020-09775-3

**Published:** 2020-08-17

**Authors:** Gireesh B. Reddy, Dylan N. Greif, Jose Rodriguez, Thomas M. Best, Harry G. Greditzer, Jean Jose

**Affiliations:** 1 Department of Orthopaedic Surgery, University of Miami Miller School of Medicine, Jackson Memorial Hospital, Miami, FL USA; 2 Department of Radiology, University of Miami Miller School of Medicine, Jackson Memorial Hospital, Miami, FL USA; 3 University of Miami Sports Medicine Institute, University of Miami Miller School of Medicine, Coral Gables, FL USA; 4 Department of Radiology, Hospital for Special Surgery, New York, NY USA

**Keywords:** COVID-19, coronavirus, diagnostic imaging, orthopedics

## Abstract

The COVID-19 pandemic holds widespread implications for global public health, economies, societies, and the practice of orthopedic surgery. As our knowledge of the transmissibility of SARS-CoV-2 and the symptomatology and management of COVID-19 expands, orthopedic surgeons must remain up to date on the latest medical evidence and surgical perspectives. While COVID-19 primarily manifests with pulmonary symptoms, cardiovascular, neurologic, and other major organ systems may also be affected and present with hallmark imaging findings. This article reviews initial and emerging literature on clinical characteristics and imaging findings of COVID-19.

**Electronic supplementary material:**

The online version of this article (10.1007/s11420-020-09775-3) contains supplementary material, which is available to authorized users.

## Introduction

Since December 2019, infections with severe acute respiratory syndrome coronavirus 2 (SARS-Cov-2), a novel betacoronavirus strain responsible for coronavirus disease 2019 (COVID-19), rapidly progressed from an isolated cluster of cases in the Hubei province of east central China to a pandemic, with significant global health and economic repercussions [4, 5, 10, 24, 25, 27, 28, 44, 58, 80, 91]. By mid-June 2020, the World Health Organization (WHO) had declared over 4.3 million people infected worldwide, with nearly 300,000 deaths. In the USA, despite unprecedented social distancing and public health measures, over 1.3 million people were infected, with more than 100,000 deaths [21]. Musculoskeletal chief complaints account for 18% of all healthcare visits in the USA [59], with nearly 23 million orthopedic procedures performed annually worldwide, of which five orthopedic procedures comprise 17% of all operations in the USA [4]. Public safety restrictions on semi-elective and elective office visits and surgical procedures during the COVID-19 pandemic have created a tremendous backlog (estimated at more than 1 million cases in 2 years) for orthopedic practices and have taken many practices to a tenuous economic precipice [4, 39].

As restrictions are eased nationally [1], it is critical that orthopedic surgeons remain aware of the clinical and radiographic findings associated with COVID-19 to best evaluate surgical patients. In addition to the widely known pulmonary symptoms, COVID-19 patients may initially present with atypical neurologic, gastrointestinal, cardiac, and musculoskeletal imaging findings (Table 1), which are more likely to be undiagnosed. We summarize the most recent literature describing the clinical and imaging findings in order to assist orthopedic surgeons in navigating a clinical and practice management landscape permanently transformed by the pandemic.

**Table 1:** Summary of reported COVID-19 most common imaging findings to date

Organ system	Imaging findings
Pulmonary manifestations	Plain radiograph (Fig. 1) • Consolidation and ground glass opacities (GGO) in a peripheral and lower lobe distribution, with predominately bilateral lung involvement • Pulmonary nodules, pleural effusions, lymphadenopathy, and lung cavitation are usually absent
	Chest CT findings based on time of illness (Figs. 2, 3, and 4) *A*. Early Stage (days 0–4) • Subpleural unilateral or bilateral GGO • Negative findings possible in minority of patients *B*. Progressive Stage (days 5–8) • Diffuse/multilobe distribution of GGO • Crazy-paving pattern (GGO with superimposed inter- and intralobular septal thickening) • Consolidations without mediastinal lymphadenopathy *C*. Peak Stage (dDays 9–-13) • Worsening GGO diffusion and crazy-paving with residual parenchymal bands • ARDS highly likely during this period *D*. Absorption Stage (dDays 14-resolution) • GGO may persist, but crazy-paving resolves • Consolidations decrease over time
	Other associated chest CT findings, Fig. 7 • Septal thickening • Pleural thickening • Pericardial effusion • Bronchiectasis • CT Halo sign • Acute pulmonary embolism (screen for deep vein thrombosis on duplex ultrasound)
Cardiovascular manifestations	Gadolinium-enhanced cardiac MRI and echocardiographs **(**Figs. 5 and 6**)** • Acute myopericarditis: curvilinear delayed enhancement in the subepicardial wall and adjacent pericardium • Acute myocardial infarction: delayed transmural enhancement within ventricle • Generalized increase in heart wall thickness • Diffuse biventricular hypokinesis • Severe left ventricular dysfunction • Biventricular myocardial interstitial edema • Pericardial effusion (mostly around the right cardiac chambers)
Musculoskeletal and neurologic manifestations	CT brain (Figs. 8 and 9) • Acute large vessel cerebral infarcts (could be thromboembolic in nature) • Acute cerebral hemorrhage • Leukoencephalopathy, including CT hypoattenuation of the bilateral cerebral hemispheric white matter and corpus callosum
	MRI (with or without IV contrast) (Figs. 10, 11, and 12) • Encephalitis with leptomeningeal enhancement • Meningoencephalitis • Guillain-Barré syndrome (GBS) • Acute ischemic stroke with frontotemporal hypoperfusion abnormalities • Intracranial hemorrhage • Cerebral venous thrombosis • Multiple sclerosis plaque exacerbation • Miller-Fisher syndrome • Posterior reversible encephalopathy syndrome (PRES) • Acute necrotizing encephalopathy (ANE) • Leukoencephalopathy with diffuse confluent white matter T2/FLAIR hyperintensities, scattered micro-hemorrhage in the corpus callosum, and posterior circulation hyperperfusion, without diffusion restriction or abnormal enhancement • Myositis
Gastrointestinal manifestations	CT and US abdomen (Figs. 13 and 14) • Small and large bowel wall thickening, due to gastroenteritis or ischemia • Bowel and mesenteric infarction and necrosis, with associated non-enhancing bowel, pneumatosis, portal venous gas, and bowel perforation • Portal vein thrombosis • Distended gallbladder containing sludge suggestive of cholestasis • Solid organ inflammation and infarction, including the pancreas (pancreatitis), liver (hepatitis), kidneys, and spleen

*CT* computed tomography

*MRI* magnetic resonance imaging

### Pulmonary Manifestations

**Fig. 1:**
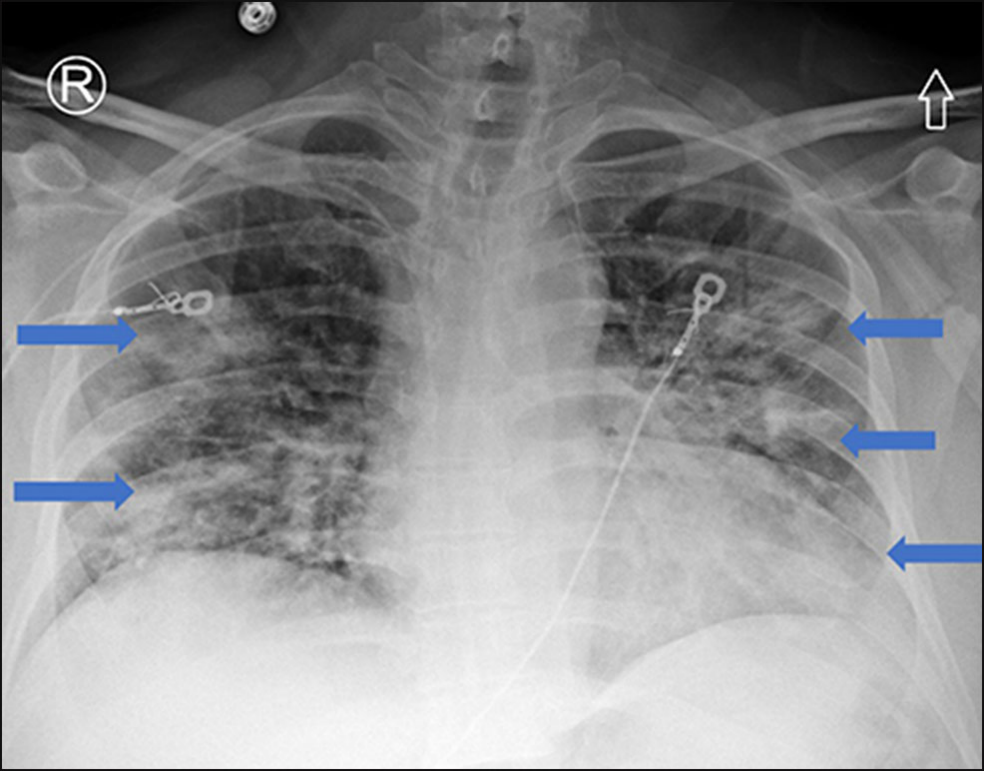
Chest X-ray findings of COVID-19 pneumonia: frontal radiograph of the chest demonstrates low lung volumes with bilateral perihilar ground glass opacities and peripheral airspace consolidations (blue arrows) in a predominately mid and lower lobe distribution.

The most common symptoms of patients presenting with COVID-19 are cough, dyspnea, and fever, while the most common reasons for admission are pneumonia and hypoxemia [12, 27, 36, 80, 91]. Approximately 14% of patients develop more severe symptoms, including acute hypoxic respiratory failure and acute respiratory distress syndrome (ARDS), while the mortality rate for patients requiring invasive mechanical ventilation is high (24.7% in New York City) [68, 81]. In a recent retrospective review of chest radiographic findings in 64 patients with COVID-19, Wong et al. reported consolidation (47%) and ground glass opacities (GGO) (33%) as the most common findings, usually in a peripheral (41%) or lower lobe (51%) distribution, with bilateral lung involvement in 50% (Fig. 1) [72]. Pulmonary nodules, pleural effusions, lymphadenopathy, and lung cavitation (thick-walled abnormal gas-filled spaces within the lung) were usually absent [16]. Chest computed tomography (CT) is the gold imaging standard for diagnosing COVID-19. In a retrospective cohort study from Wuhan, including some of the earliest diagnosed patients, CT scans were reviewed sequentially from prior to symptom onset to 3 weeks after onset [72]. The authors found that even before symptom onset, CT scans demonstrated unilateral GGO that progressed to bilateral diffuse GGO, with or without consolidation [57, 72].

**Fig. 2:**
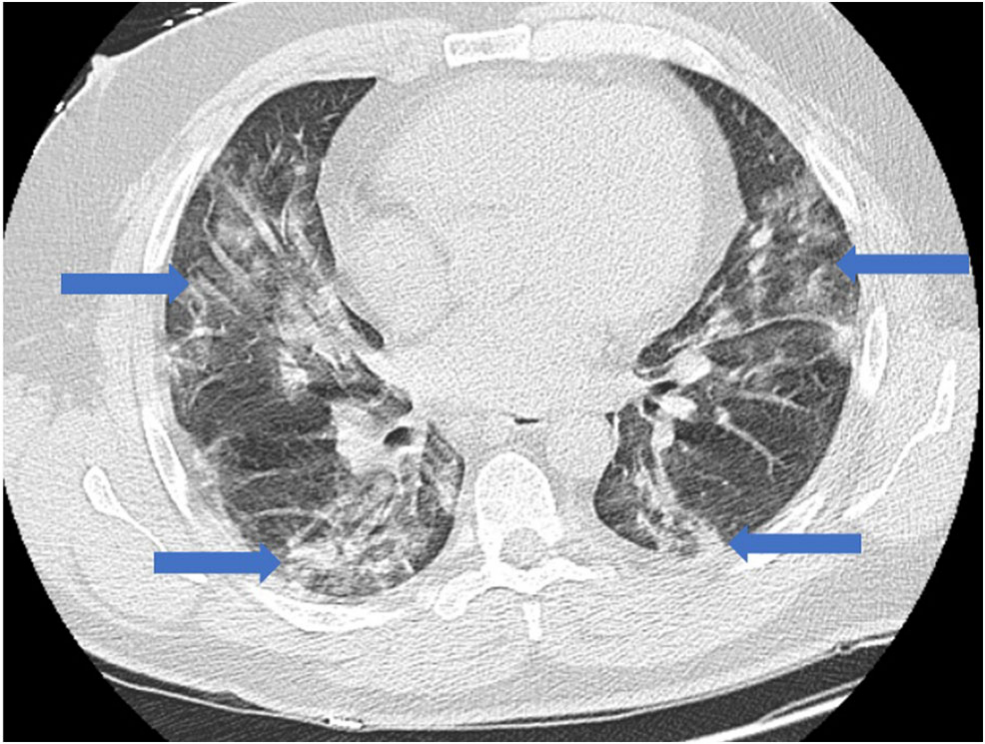
Early stage COVID-19 (0–4 days): Axial CT scan of the chest in a 52-year-old man with COVID-19 predominantly demonstrates peripheral ground glass opacities (blue arrows).

**Fig. 3:**
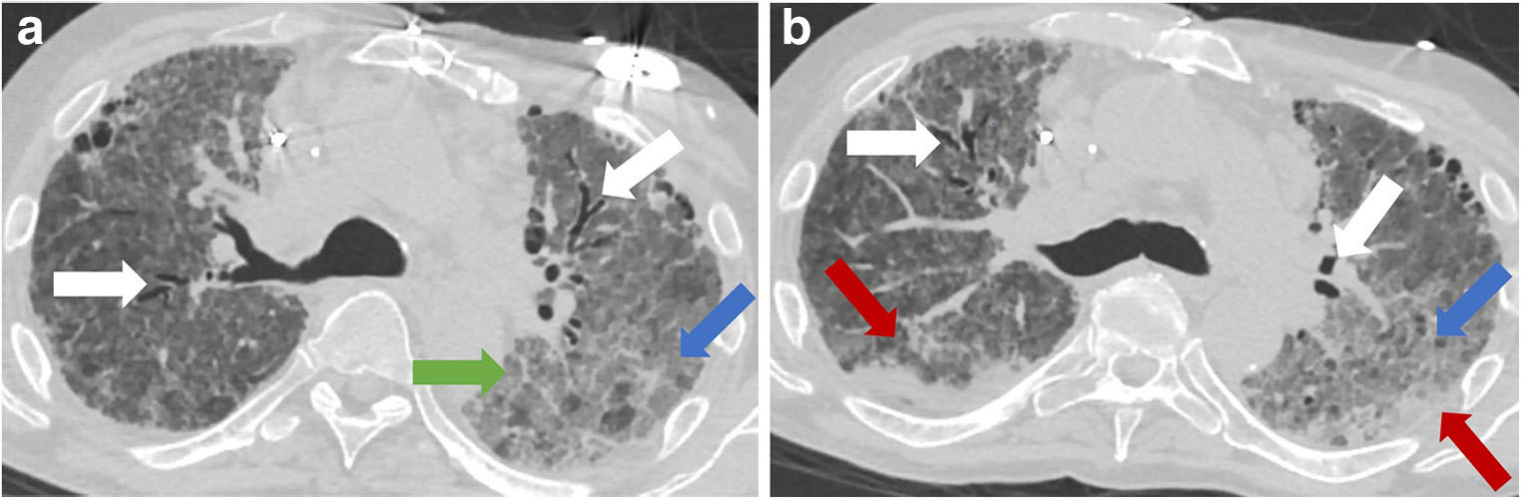
Progressive stage COVID-19 (5–8 days): Axial CT of the chest demonstrates diffuse distributionof GGO (blue arrows in A and B), crazypaving pattern (green arrow in A), consolidations (red arrows in B), and bronchiectasis (white arrows).

**Fig. 4:**
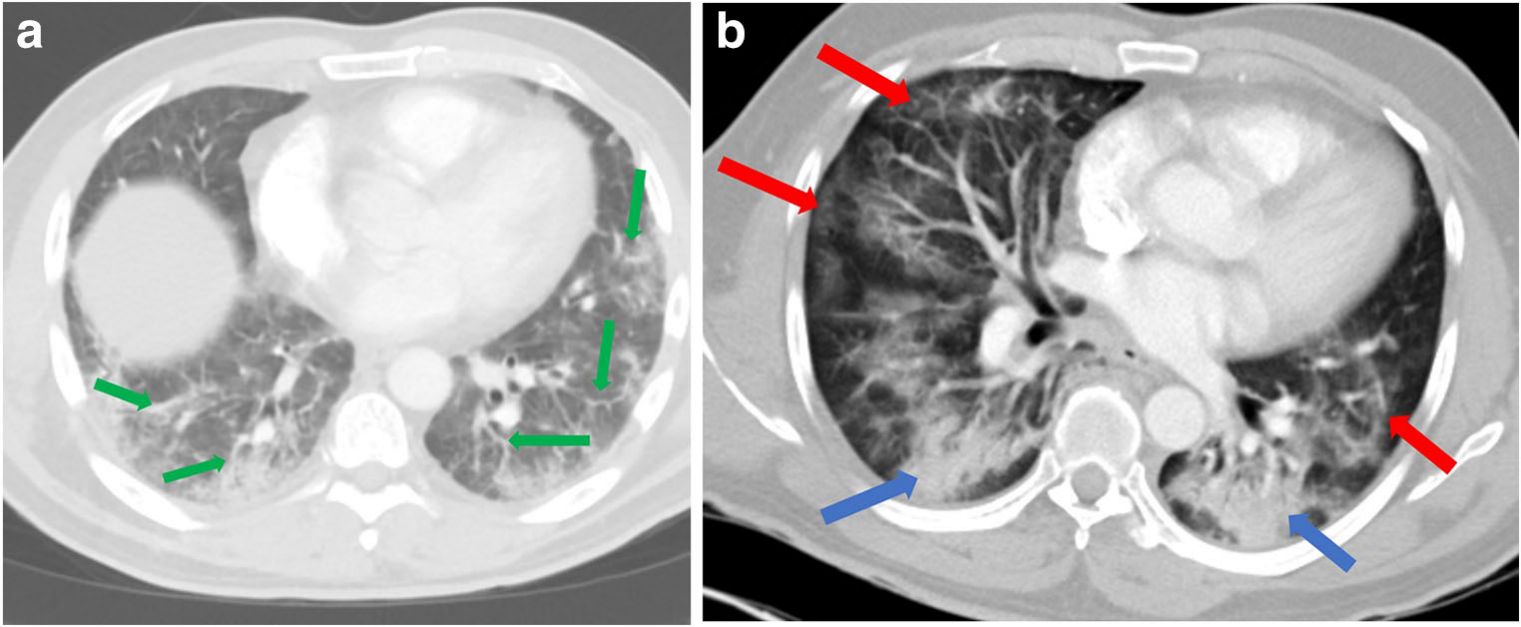
Peak stage COVID-19 (9 to 13 days): **a** Axial CT scan of the chest in a positive 49-year-old man with COVID-19 demonstrates ground glass opacities (GGO) in a multilobe distribution (red arrows) and bibasilar consolidations (blue arrows). **b** Diffuse crazy-paving pattern (GGO with superimposed inter- and intralobular septal thickening), consolidations, and residual parenchymal bands (green arrows).

Early reports from China described the most common imaging findings on CT as GGO (56.4%) and bilateral patchy shadowing (51.8%) [27]. In another recent review, Pan et al. correlated time course of lung changes on CT scans with COVID-19 disease progression [61]. In the “early stage” of the disease (0 to 4 days after onset of symptoms), GGOs in subpleural locations unilaterally or bilaterally were observed (Fig. 2). During the “progressive stage” (days 5 to 8), CT scans demonstrated multilobe distribution of diffuse GGOs and crazy-paving pattern (GGOs with superimposed inter- and intralobular septal thickening), and consolidation was observed, without mediastinal lymphadenopathy (Fig. 3) [70]. In the “peak stage” (9 to 13 days), consolidations became denser, with worsening diffuse GGOs, crazy-paving, and residual parenchymal bands (Fig. 4). If patients clinically improved, they entered the “absorption stage” (usually more than 14 days after symptom onset). In this stage, GGOs persisted, but the crazy-paving resolved, and consolidations improved. If the patient worsened, with increased oxygen requirements, need for more invasive ventilation, and other intensive care unit (ICU) care, a transition to a denser alveolar consolidation pattern on radiographic imaging was noted. At this point, acute respiratory distress syndrome was likely to occur, and a low clinical threshold was necessary for transfer to an ICU for advanced respiratory support [70].

There is a characteristic change in imaging of the chest, not only temporally from symptom onset but also with increasing disease severity. In a retrospective study of 83 patients, patients with more severe manifestations of COVID-19 had higher thin section CT score, incidence of consolidation, mediastinal lymph node enlargement, septal thickening, pleural effusion, and pericardial effusion than those with less severe presentations [47, 77]. Salehi et al. reviewed the imaging findings of 919 patients and corroborated this progression of severity [70]. Other uncommon findings that occur later in the progression of COVID-19 may also include bronchiectasis, pleural thickening, enlargement of intralesional pulmonary vessels greater than 3 mm in diameter, cavitation, or CT halo signs (GGOs surrounding a pulmonary nodule or mass) [66, 70]. Finally, as mentioned, disease resolution is seen with the gradual disappearance of consolidative opacities and decreased number of lesions and/or involved lobes. A recent meta-analysis of 68 articles [42] found that chest CT has a sensitivity and specificity of 94% and 37%, respectively, in detecting COVID-19 [3, 22]. Imaging characteristics of COVID-19 are distinctive enough that Chinese and American radiologists were able to differentiate COVID-19 (*n* = 219) from respiratory viral pneumonia (*n* = 205) when presented with respective imaging [6].

### Cardiovascular Manifestations

**Fig. 5:**
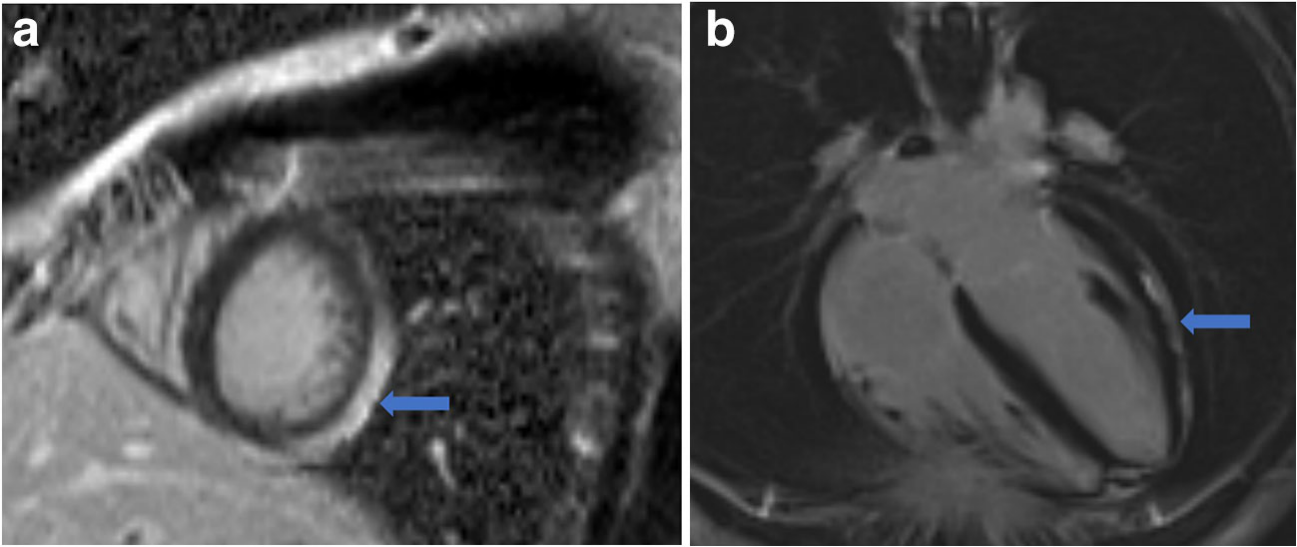
Myopericarditis in a patient with chest pain, ST elevation, and elevated cardiac troponins: **a** Two chamber view and **b** four chamber view post-contrast cardiac MRI demonstrate curvilinear delayed enhancement in the subepicardial lateral mid and apical wall of the left ventricle and the adjacent pericardium (blue arrows).

**Fig. 6:**
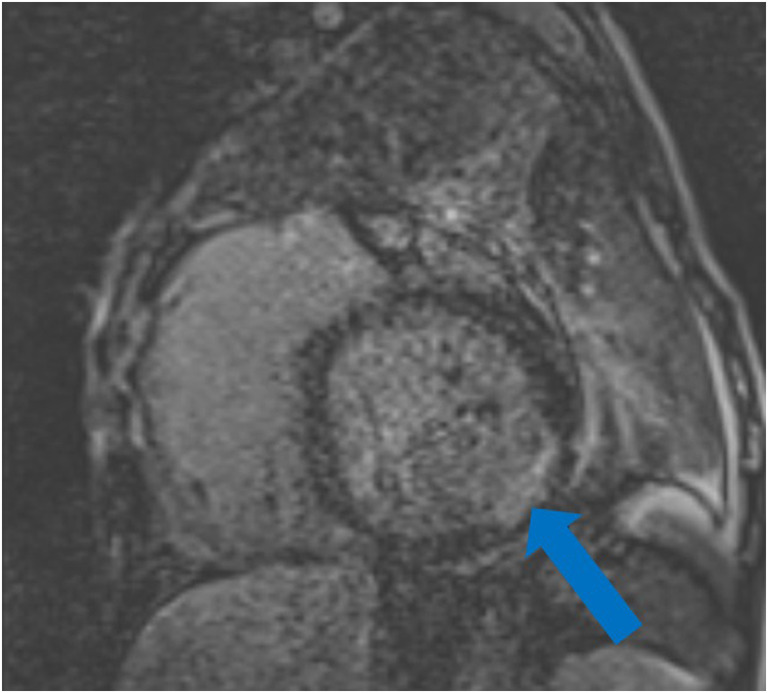
Myocardial infarction (MI) in a 56-year-old man: Two chamber post-contrast cardiac MRI shows transmural delayed enhancement consistent with MI involving the inferolateral base of the left ventricle.

Early reports from Italy and China indicated that although pulmonary diseases including ARDS and diffuse pneumonia comprise the predominant lethal complications of COVID-19, patients have also presented with or developed significant cardiac signs and symptoms [50]. In one of the first series of COVID-19 patients from Wuhan, Huang et al. [36] found that five of 41 (12%) had some form of acute cardiac injury, determined either by elevated cardiac biomarkers above the 99th percentile or new abnormalities/arrhythmias on electrocardiography or echocardiography as seen in patients with myopericarditis (Fig. 5) or myocardial infarction (Fig. 6) [50, 89]. A larger retrospective cohort study of 187 patients with laboratory positive COVID-19 demonstrated that 27.8% patients had some form of myocardial injury as defined by elevated cardiac biomarkers. Mortality rates were nearly doubled in those with preexisting cardiovascular disease (CVD) (13.33% vs. 7.62%), but mortality rates increased dramatically if acute cardiac injury occurred during hospitalization (37.5% for those without concomitant CVD and 69.44% for those with concomitant CVD) [29]. A study of 416 patients with COVID-19 found a 19.7% incidence of cardiac injury; these patients had worse laboratory markers, a higher proportion requiring advanced airway management, and higher mortality rate from symptom onset (hazard ratio (HR): 4.26 [95% CI: 1.92–9.49]) [73]. A case report from Italy described a COVID-19 patient without a CVD history who presented with electrocardiographic and biomarker changes indicative of acute cardiac injury [38]. Transthoracic echocardiography (TTE) and gadolinium-enhanced cardiac magnetic resonance imaging (Gd-cardiac MRI) demonstrated increased wall thickness, diffuse biventricular hypokinesis, severe left ventricular dysfunction, biventricular myocardial interstitial edema (on short-tau inversion and T2-mapping sequences), acute myocarditis, and pericardial effusion, especially around the right cardiac chambers.

The above suggests that COVID-19 may be related to cardiac injury of undetermined pathophysiology. Cardiac injury in the context of viral infections has been studied in the past. In a self-controlled case series involving 365 admissions in Ontario, Canada, laboratory-confirmed respiratory viral infections were associated with 6.05% increase in the incidence of acute myocardial infarction within 7 days of viral detection [45]. Viral respiratory tract infections may lead to complications such as acute coronary syndrome (ACS) or venothrombotic events (VTEs) secondary to immune system hyperactivation and generation of a thrombogenic state [18, 43].

### Thromboembolic Complications

**Fig. 7:**
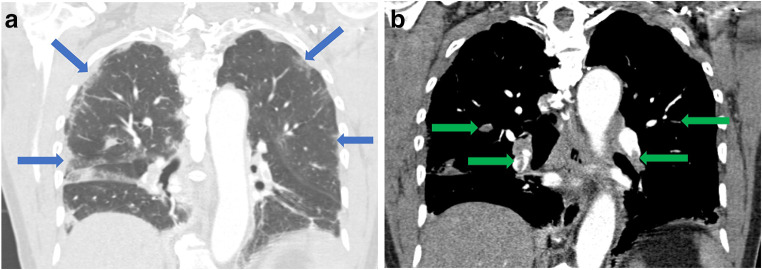
Early stage COVID-19 (0 to 4 days) with acute pulmonary embolism: **a** Coronal CT scan of the chest (lung window) in a 79-year-old man with COVID-19 demonstrates peripheral ground glass opacities in subpleural distribution (blue arrows). **b** Coronal CT scan of the chest with IV contrast (soft tissue window) demonstrates filling defects within the right and left pulmonary arteries (green arrows), indicating acute pulmonary embolism.

Global reports of COVID-19-associated coagulopathy with multifocal thromboembolic disease are rapidly growing and include pulmonary emboli, limb ischemia, and cerebral infarct; often associated with poorer prognosis [19, 51, 54, 75, 87, 88]. Several European studies have demonstrated higher rates of VTE in the COVID-19 population [32, 49]. In a study of 150 COVID-19 ICU patients, nearly 64 (43%) had a thrombotic event, including pulmonary embolism (Fig. 7) and continuous renal replacement therapy (CRRT) circuit clotting [32]. In a study of 48 COVID-19 ICU patients at two institutions, an alarming 85% were positive for DVTs, despite almost all being on prophylactic anticoagulation [67]. Another study of 26 ICU patients screened for DVTs using duplex ultrasonography found that 69% were positive, with 56% positive despite being on therapeutic anticoagulation [48]. Up to 30% of COVID-19 patients with pulmonary symptoms were diagnosed with acute pulmonary embolus on pulmonary CT angiogram over a 1-month period in a tertiary care center and all with higher levels of D-dimer (2660 μg/L) and C-reactive protein (CRP) than usually encountered, suggesting an independent association between D-dimer level and disease severity [46, 65]. Antiphospholipid antibodies in the setting of multifocal cerebral infarct have also been observed in COVID-19 patients [88]. While noted in other infections, antiphospholipid antibodies have not been associated with thrombotic events in these circumstances [71, 88].

### Neurologic Manifestations

COVID-19 musculoskeletal and neurologic manifestations are being reported with increased frequency, particularly in patients with more severe respiratory disease, indicating coronavirus neurotropism possibly directly related with higher viral loads, which are now detectable in cerebrospinal fluid [20]. Angiotensin-converting enzyme 2 (ACE-2) receptors may be responsible for COVID-19 cerebral involvement via entry through the cribriform plate [56].

**Fig. 8:**
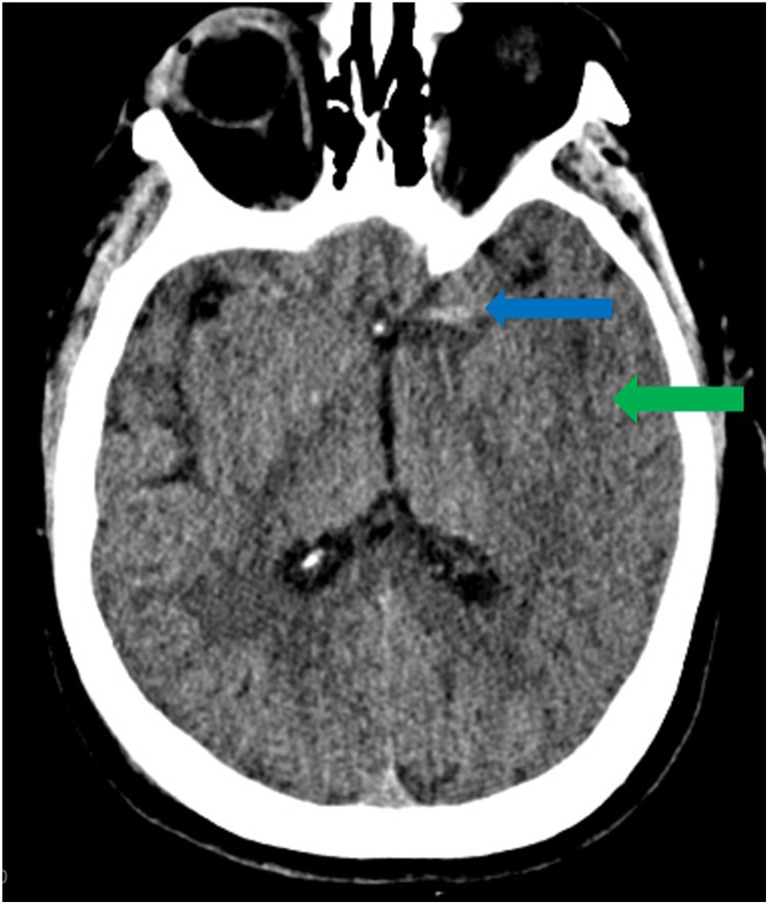
Acute thromboembolic infarction in a 57-year-old: Axial non-contrast CT scan demonstrates left hyperdense middle cerebral artery (MCA) sign (blue arrow), with increased density in the M1 segment of the left MCA, consistent with acute thromboembolic occlusion. There is subtle loss of gray-white matter differentiation, hypodensity, and engorgement along the left insular ribbon and temporal operculum (loss of insular ribbon sign), reflecting acute infarction (green arrow).

**Fig. 9:**
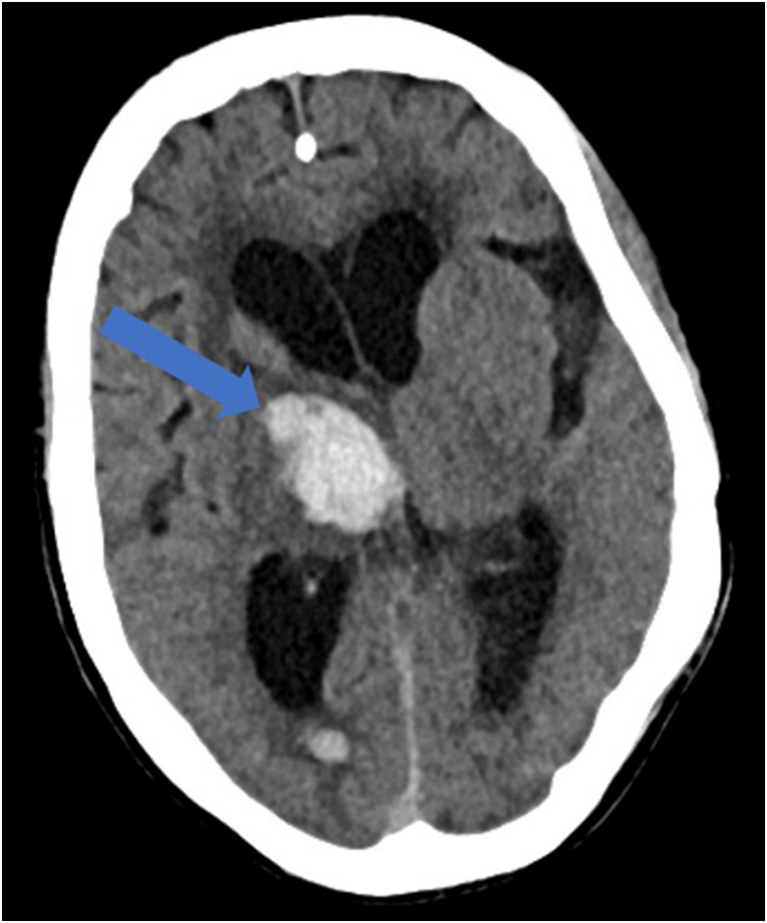
Intracranial hemorrhage in a 60-year-old: Axial non-contrast CT scan demonstrates intraparenchymal hemorrhage within the right basal ganglia and thalamus, with associated intraventricular extension, dilatation, and midline shift (blue arrow).

Reportedly, up to 84% of ICU COVID-19 patients demonstrate neurologic symptoms [31, 41]. Neuroimaging findings beyond those of acute infarction, hemorrhage, and vessel thrombosis include meningoencephalitis and acute necrotizing encephalopathy (ANE). There is mounting evidence of increased leptomeningeal enhancement, fluid-attenuated inversion recovery (FLAIR) cortical signal abnormalities, cortical diffusion restriction, and cortical blooming artifact, suggesting either infectious, autoimmune, or critical illness-related encephalitis, hypoxia, hypoglycemia, and seizure [41]. In a limited series over a 2-week period in March 2020 at a single institution, five patients under the age of 50 years suffered large vessel arterial cerebral infarct (with preferential involvement of the middle cerebral artery), a dramatic increase compared with a pre-COVID-19 average of 0.79 strokes per 2 weeks in patients younger than 50 years (Fig. 8) [60]. In a study by Mao et al. of 214 patients with COVID-19, 36.4% had neurologic symptoms, including acute ischemic stroke, intra-cranial hemorrhage (Fig. 9), impaired consciousness, and skeletal muscle injury defined as pain with elevated serum creatinine kinase levels [53, 64].

**Fig. 10:**
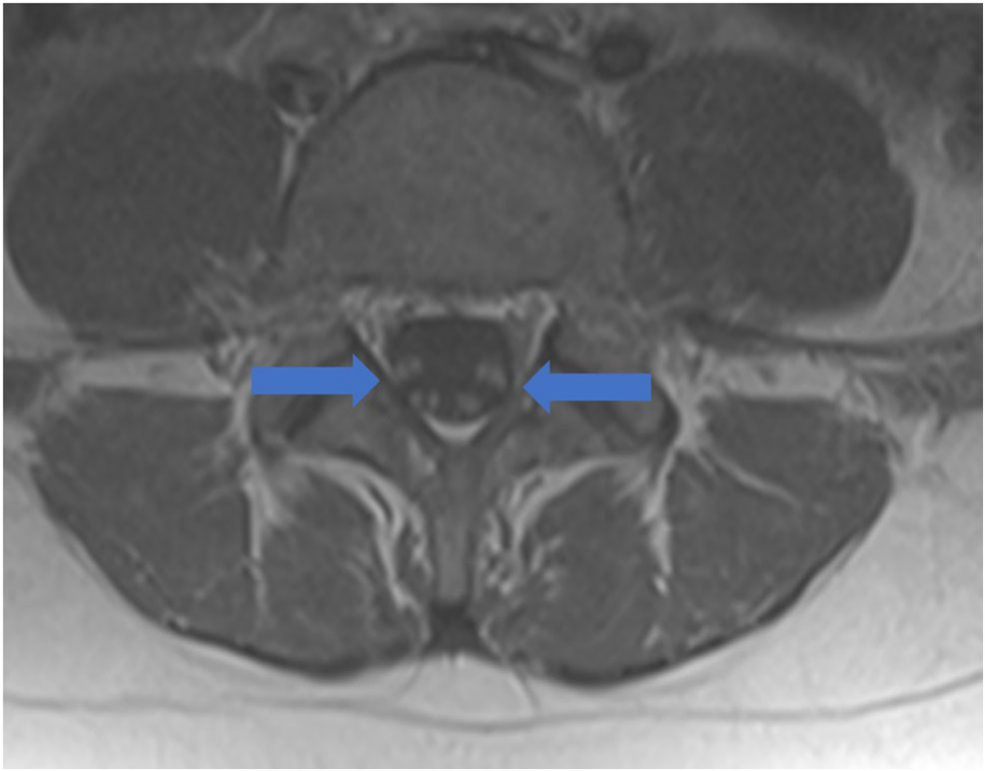
Guillain-Barré syndrome: Axial post-contrast T1 MRI of lumbar spine demonstrates diffuse enhancement without enlargement of the ventral and dorsal nerve roots (blue arrows).

**Fig. 11:**
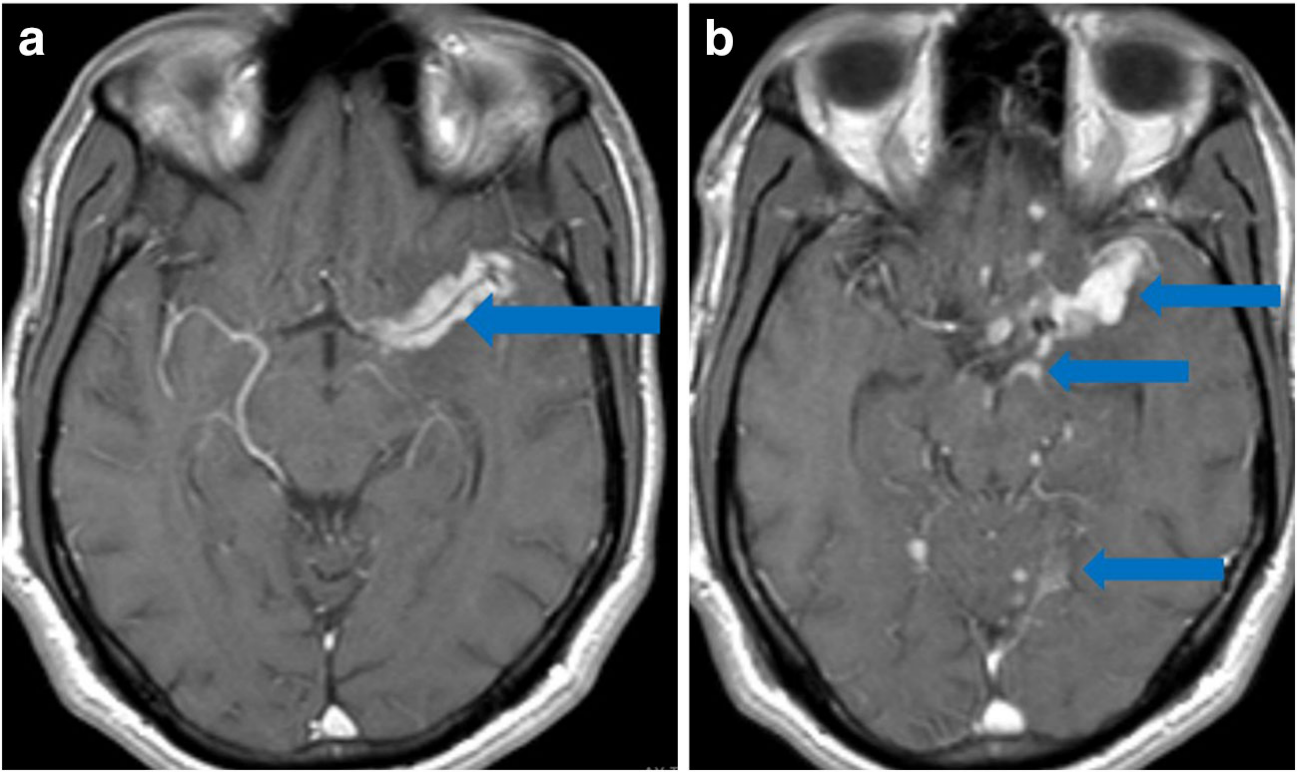
Leptomeningeal enhancement and encephalitis in a 39-year-old with neurologic impairment: **a** Axial post-contrast MRI demonstrates leptomeningeal enhancement along the left middle cerebral artery (MCA) distribution (blue arrow). **b** Axial post-contrast MRI demonstrates leptomeningeal enhancement along the left MCA, as well as the basal cisterns and superior cerebellum (blue arrows).

From Italy, of an estimated 1200 COVID-19 patients admitted over 1 month, five developed rapidly progressive Guillain-Barré syndrome (Fig. 10) that began with lower extremity paresis that progressed to flaccid tetraparesis and tetraplegia [76]. Mahammedi et al. reported that 108 of 725 (15%) consecutive hospitalized COVID-19 patients had neurologic symptoms requiring neuroimaging in Italy, and of those, 64 (59%) had altered mental status, and 34 (31%) had ischemic stroke; 47% of those patients had acute neuroimaging abnormalities that included acute ischemic stroke, intracranial hemorrhage (possibly due to cerebrovascular endothelial rupture), cerebral venous thrombosis, multiple sclerosis plaque exacerbation, encephalopathy, Guillain-Barré syndrome, Miller-Fisher Syndrome, and posterior reversible encephalopathy syndrome [7, 52]. In an observational study of 58 encephalopathic COVID-19 patients with ARDS, 38 had upper motor neuron signs, while eight of 13 who underwent MRI demonstrated leptomeningeal enhancement (Fig. 11), two of 13 had focal acute ischemic strokes, and 11 of 11 displayed frontotemporal hypoperfusion abnormalities on profusion studies [31]. Anosmia, possibly related to ACE-2 involvement, has also been reported [33].

### Musculoskeletal Manifestations

**Fig. 12:**
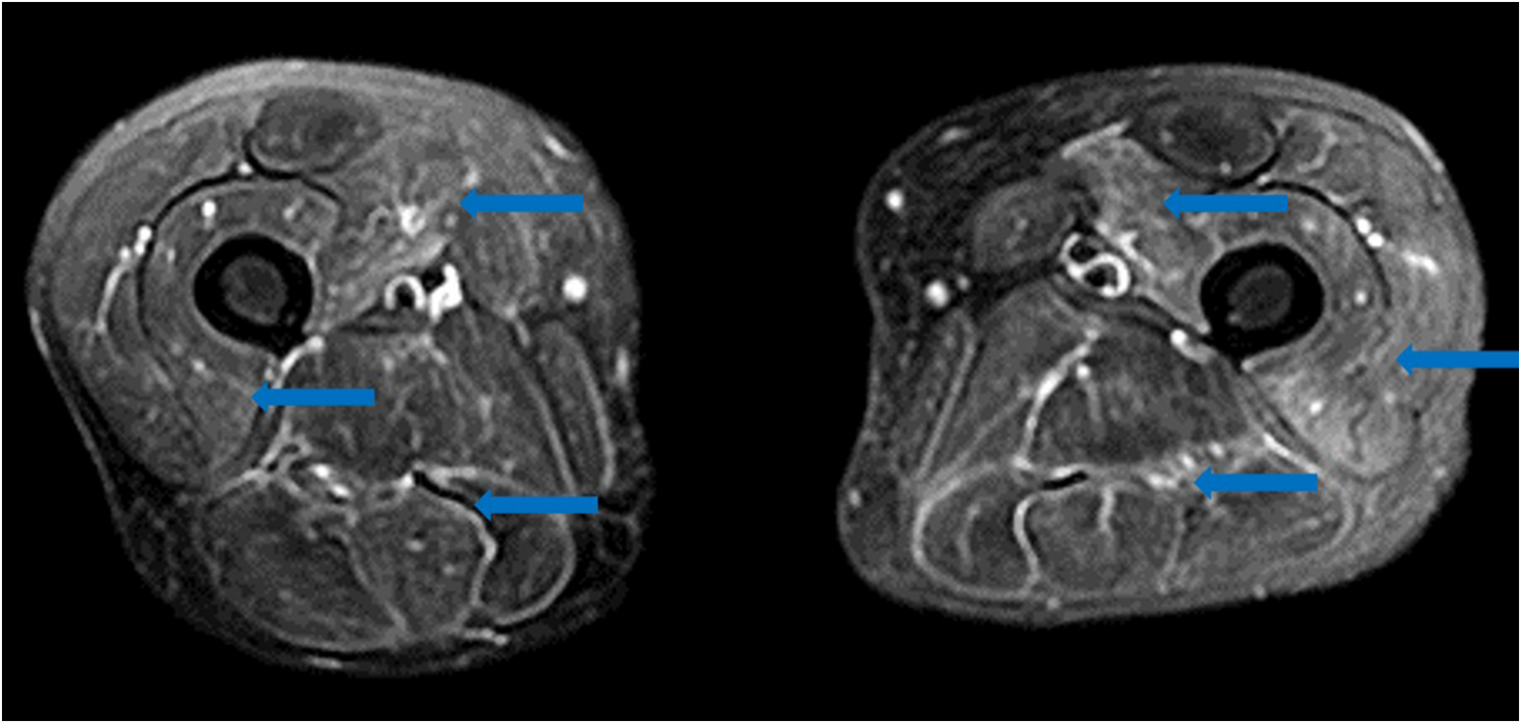
Axial fluid sensitive MRI of the thighs demonstrates diffuse multicompartment intramuscular high T2 signal, indicative of edema and myositis.

Limited reports of COVID-19 isolated musculoskeletal manifestations are currently available. Similar low incidence case series of critical illness myopathy or myositis (Fig. 12) [55] and critical illness polyneuropathy following Middle East respiratory syndrome (MERS) and severe acute respiratory syndrome (SARS) have also been reported, at times with an association with concurrent myocarditis [9, 14, 74]. In all of these cases, patients typically present with myalgia and muscle weakness, with rhabdomyolysis and elevated creatine kinase (CK) levels representing more severe manifestations [27, 78, 86]. Elevated CK levels are associated with increased rates of mortality and interstitial pneumonia in COVID-19 patients [90].

### Cytokine Storm

Evidence suggests that some patients with severe COVID-19 might have a cytokine storm syndrome, which can be seen by testing for serum cytokine levels [37]. Recently, ANE, a rare complication of viral infections related to intracranial cytokine storms that result in blood-brain barrier breakdown without direct viral invasion or demyelination, has been reported as a result of COVID-19 [65]. The most common findings of ANE include hypoattenuating lesions on CT and T2/FLAIR hyperintense lesions on MRI, in a bilateral symmetric multifocal distribution predominantly involving the thalami and to a lesser extent the brain stem, cerebral white matter, and cerebellum. In addition, findings of leukoencephalopathy, including CT hypoattenuation of the bilateral cerebral hemispheric white matter and corpus callosum, as well as MRI diffuse confluent white matter T2/FLAIR hyperintensities, scattered micro-hemorrhage in the corpus callosum, and posterior circulation hyperperfusion without diffusion restriction or abnormal enhancement have been reported [69]. It is uncertain if COVID-19-related white matter injury findings are the result of ICU anesthesia-related toxicity, COVID-19-associated cytokine release syndrome, or COVID-19-related endotheliitis with thrombotic microangiopathy [69].

Beyond neurologic manifestations, cytokine storm also has pulmonary manifestations, namely, pulmonary and interstitial damage caused by nonspecific inflammatory cell infiltration [83]. For COVID-19, in addition to the previously described radiographic findings, this syndrome may lead to the development of bilateral pneumonia or ARDS much faster in thus subgroup of patients, as well increased rates of ICU admission, mechanical ventilation, and subsequent mortality [83].

Even without pulmonary or neurologic manifestations, cytokine storm can lead to multi-organ system failure, which explains reports of elevated liver enzymes, creatine, and other important markers in COVID-19 patients who do not present with the above manifestations [83]. This is due to the systemic exposure to large amounts of pro-inflammatory cytokines that leads to the immune system “attacking” the body, particularly interstitial and parenchymal spaces [17]. Vascular compromise due to extensive endothelial damage is also possible [79].

### Gastrointestinal and Hepatic Manifestations

**Fig. 13:**
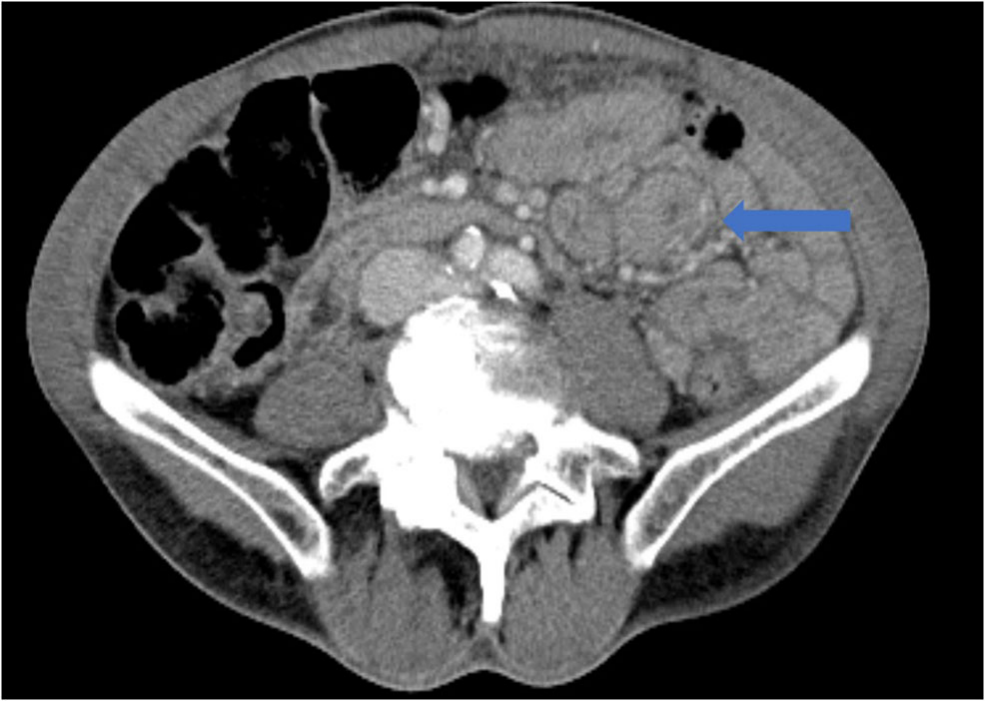
Axial post-contrast CT scan demonstrates diffuse bowel wall thickening of the distal duodenum and jejunum (blue arrow), indicative of gastroenteritis.

Though not as severe, gastrointestinal symptoms have been reported in up to 20% of COVID-19 patients [15]. This may be due to the increased gastrointestinal wall permeability to viral pathogens, which promote malabsorption by infected enterocytes [26]. The most common symptoms include diarrhea, abdominal pain, or vomiting, though preliminary data suggests that patients with these symptoms tend to have an improved prognosis independent of patient age or sex [2]. Constipation, melena, and anorexia have also been reported but less commonly [34, 85]. More severe manifestations of gastroenteritis can be seen on CT scan and can be the only presenting symptom (Fig. 13) [30, 62]. Patients can have pertinent laboratory findings, namely, elevated lipase and/or alkaline phosphatase levels, which are associated with poor prognosis as they reflect a greater systemic inflammatory response [2].

**Fig. 14:**
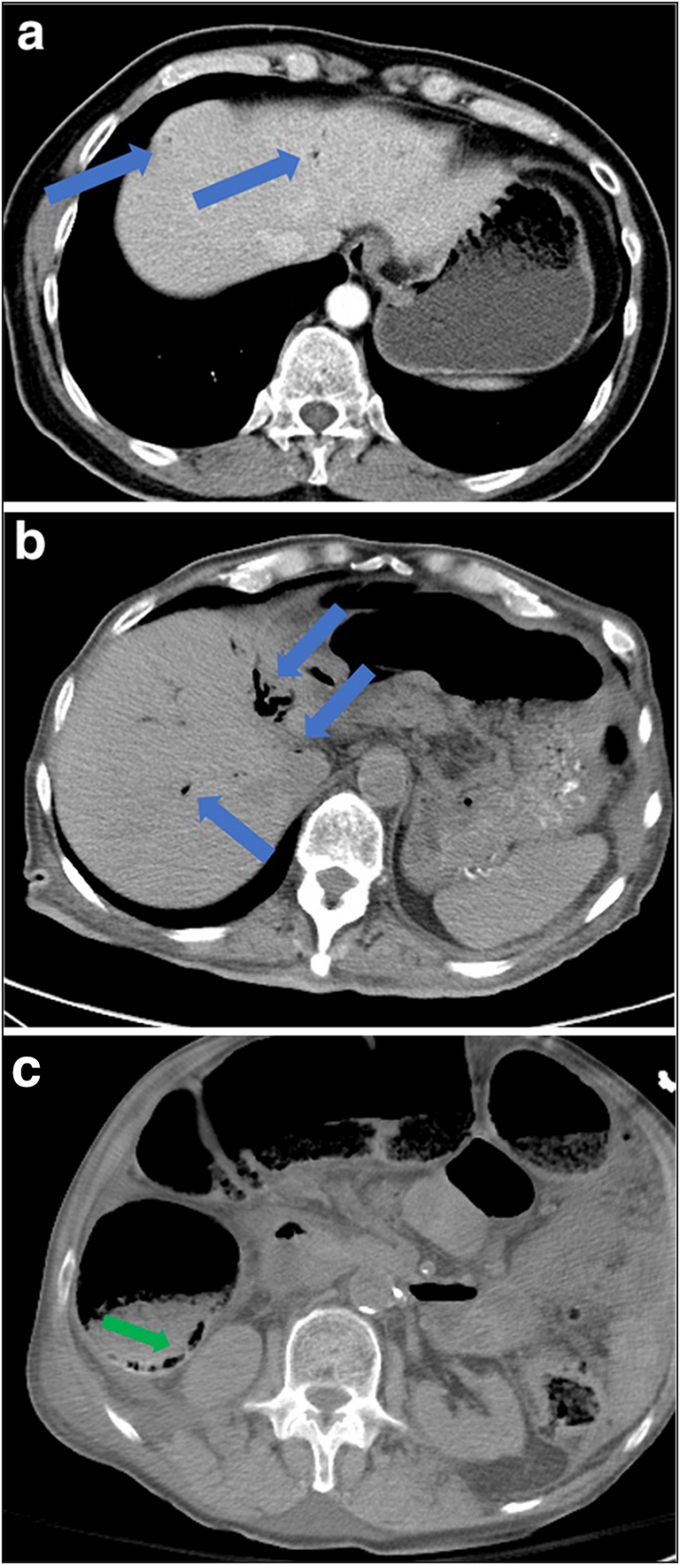
**a, b** Axial CT scan images demonstrate intraluminal air foci in the main portal venous systemic vessels and peripherally in the intrahepatic vessels (blue arrows). **c** Axial CT scan demonstrating a pneumatosis intestinalis (green arrow).

The most common abdominal imaging findings in COVID-19 ICU patients include small and large bowel wall thickening, non-enhancing bowel, pneumatosis, portal venous gas, and bowel perforation, all related to bowel and mesenteric ischemia, infarction, and necrosis (Fig. 14) [11]. Portal vein thrombosis and distended gallbladder containing sludge suggestive of cholestasis have also been observed. In addition, evidence of inflammation and infarction in other solid organs, including the pancreas, liver, kidneys, and spleen, have been reported, particularly in ICU COVID-19 patients. Although the exact pathophysiology is uncertain, these findings are thought to be multifactorial in origin, resulting from a combination of direct SARS-CoV-2 infection and indirect systemic derangements associated with critical illness, including small vessel thrombosis related to ICU hypercoagulopathy and nonocclusive ischemia related to an ICU hyperinflammatory effect [11, 35].

Though preliminary in nature, current findings may suggest that SARS-CoV-2 can be detectable in both hepatic tissue and cholangiocytes due to ACE-2 expression [13]. Elevated liver enzymes have been found in blood samples of COVID-19 patients, but it is important to note that elevated liver enzymes do not necessarily mean liver damage is present [23]. Imaging is currently not indicated for evaluation of hepatic pathology in COVID-19 patients, but hepatic steatosis and mild lobular and portal inflammation may be seen. Whether these are indeed due to COVID-19 or the consequence of drug-induced hepatic injury is still unknown [82].

### Renal Dysfunction

Recent studies suggest that COVID-19 may cause acute renal failure, as both podocytes and proximal convoluted tubular cells express certain genes (ACE-2 and transmembrane serine protease 2 [TMPRSS2]) that increase the host viability for SARS-CoV-2 [63]. The viral cytopathic effect on these cells has been linked as the cause of acute renal failure, which is secondary to ARDS as one of the more common fatal presentations in patients with COVID-19 [82]. Separately, a case report found that patients who developed rhabdomyolysis throughout the course of their disease are also at risk for acute renal failure, with manifestations of lower extremity pain and fatigue (separate from generalized myositis) followed by rising plasma creatine level [40].

Renal imaging studies currently have little to no diagnostic role in COVID-19 patients, though one would theoretically expect to note increased parenchymal echogenicity via ultrasound [8]. In fact, it is more important to keep in mind that while COVID-19 patients primarily present with respiratory findings, acute renal injury should be suspected until proven otherwise, as contrast-induced nephropathy secondary to contrast-enhanced CT or MRI imaging studies may exacerbate already present renal damage secondary to COVID-19 [84].

Clinical and basic science research, as well as national public health guidance, regarding COVID-19 is rapidly and continuously evolving. It has been described as a novel, “once-in-a-century” disease. Along with our medicine and public health colleagues, orthopedic surgeons and orthopedic care providers should remain up to date on the latest COVID-19 peer-reviewed evidence, not only to better respond to changes in our clinical practice and to accurately counsel surgical patients but also to ultimately practice safe and efficacious surgery for patients in a new and changing clinical environment. In addition to the widely known pulmonary symptoms, COVID-19 may affect the neurologic, gastrointestinal, cardiac, and musculoskeletal systems (Table 1). Musculoskeletal issues remain underrecognized at this point, which may be due to the low prevalence of this disease at this time. Overall, it is imperative that all healthcare professionals have a broader understanding of all of the possible clinical and imaging manifestations of this global pandemic to improve patient and community outcomes.

## Electronic Supplementary Material

ESM 1 (PDF 1224 kb)

ESM 2 (PDF 1224 kb)

ESM 3 (PDF 1224 kb)

ESM 4 (PDF 1224 kb)

ESM 5 (PDF 1224 kb)

ESM 6 (PDF 1224 kb)
